# Insights into the pH-dependent, extracellular sucrose utilization and concomitant levan formation by *Gluconobacter albidus* TMW 2.1191

**DOI:** 10.1007/s10482-020-01397-3

**Published:** 2020-03-04

**Authors:** Frank Jakob, Clara Gebrande, Regina M. Bichler, Rudi F. Vogel

**Affiliations:** Lehrstuhl für Technische Mikrobiologie, Technische Universität München, Gregor-Mendel-Straße 4, 85354 Freising, Germany

**Keywords:** *Gluconobacter*, Sucrose, pH, Levansucrase, Levan

## Abstract

Many bacteria and archaea produce the polydisperse fructose polymer levan from sucrose upon biofilm formation via extracellular levansucrases (EC 2.4.1.10). We have investigated levansucrase-release and -activities as well as molecular size of the levan formed by the acetic acid bacterium *Gluconobacter albidus* TMW 2.1191 at varying environmental pH conditions to obtain insight in the ecological role of its constitutively expressed levansucrase and the produced levan. A buffer system was established enabling the recovery of levansucrase-containing supernatants from preincubated cell suspensions at pH 4.3–pH 5.7. The enzyme solutions were used to produce levans at different pH values and sucrose concentrations. Finally, the amounts and size distributions of the produced levans as well as the corresponding levansucrase activities were determined and correlated with each other. The data revealed that the levansucrase was released into the environment independently of its substrate sucrose, and that more levansucrase was released at pH ≥ 5.0. The glucose release and formation of high molecular weight levans (> 3.5 kDa) from 0.1 M initial sucrose was comparable between pH ~ 4.3–5.7 using equal amounts of released levansucrase. Hence, this type of levansucrase appears to be structurally adapted to changes in the extracellular pH and to exhibit a similar total activity over a wide acidic pH range, while it produced higher amounts of larger levan molecules at higher production pH and sucrose concentrations. These findings indicate the physiological adaptation of *G. albidus* TMW 2.1191 to efficient colonisation of sucrose-rich habitats via released levansucrases despite changing extracellular pH conditions in course of acid formation.

**Electronic supplementary material:**

The online version of this article (10.1007/s10482-020-01397-3) contains supplementary material, which is available to authorized users.

## Introduction

Bacterial levansucrases (EC 2.4.1.10) are extracellular enzymes that catalyze the synthesis of the β-2,6-linked fructose polymer levan. These enzymes use the energy of the glycosidic bond of their substrate sucrose for fructose polymerization while glucose is continuously released. If water is used as acceptor instead of a growing fructose polymer chain, sucrose is hydrolyzed by levansucrases (Öner et al. 2016; Velázquez-Hernández et al. 2009). Moreover, some levansucrases exhibit an intrinsic exolevanase activity leading to the degradation of the levan molecules (Méndez-Lorenzo et al. 2015). The released glucose (and fructose from potential hydrolysis reactions) can be used for metabolic purposes. Levansucrases are abundant among bacteria and archaea (Öner et al. 2016), enable sucrose utilization (Arrieta et al. 1996) and contribute to biofilm formation of plant-associated *Bacillus subtilis*, *Erwinia amylovora* or *Pseudomonas syringae* (Dogsa et al. 2013; Koczan et al. 2009; Laue et al. 2006). In sucrose-containing foods like (sweetened) sourdough breads, kefir or natto, levan is produced by indigenous starter cultures, e.g. *Lactobacillus* spp., *Gluconobacter* spp. or *Bacillus subtilis* (Natto) (Fels et al. 2018; Jakob et al. 2012a; Korakli et al. 2003; Semjonovs et al. 2016; Shih et al. 2005; Tieking et al. 2005; Tieking and Gänzle 2005; Xu et al. 2006). The macromolecular or rather hydrocolloid properties of levan in aqueous solution mainly depend on its molecular weight and can be additionally influenced by its branching degree (at position *O1*) and polydispersity (Hundschell et al. 2019, 2020; Jakob et al. 2012b, 2013). However, it is unknown, which and if a certain amount, specific size and composition of levan is essential for survival or assertiveness of the producer strains. As levansucrases are active in the extracellular environment, they are exposed to multiple continuously changing reaction conditions, e.g. in terms of the available substrate concentration, pH or temperature. Therefore and because of the great diversity of levansucrases from diverse microbial sources (Velázquez-Hernández et al. 2009), levan can be mainly composed of short-chain fructooligosaccharides (degree of polymerization of 3–10) (Támbara et al. 1999), or in other cases predominantly of high molecular weight molecules exhibiting an averaged molecular weight > 10^8^ Da (Jakob et al. 2013; Ua-Arak et al. 2017a). Levansucrases are abundant in acetic acid bacteria (*Acetobacteraceae*) and strictly present in one gene copy in all strains of different species within the genus *Gluconobacter* (Jakob et al. 2019), which typically occur in sugary, sucrose-rich environments and are specialized in glucose oxidation to gluconic acid via membrane-bound dehydrogenases (Deppenmeier and Ehrenreich 2009). Nothing is still known about the release of *Gluconobacter* levansucrases and if or to which extent their extracellular activity and product specificity are influenced by changing pH conditions in course of acid formation from e.g. naturally present sucrose. As previous studies revealed that the pH is crucial for the hydrocolloid properties, amount and the size distributions of the globular high molecular weight levan molecules produced by the water-kefir isolate *Gluconobacter albidus* TWM 2.1191 (Hundschell et al. 2020; Ua-Arak et al. 2017a), we wanted to investigate its possible physiological adaptation to extracellular sucrose consumption via released levansucrases despite changing pH conditions. For this purpose, a buffer system for the recovery of its levansucrase at different pH values and for the cell-free production of levan at different pH values and sucrose concentrations was established. The obtained data about levan amounts and sizes should finally be correlated with the volumetric activities of the levansucrases to get deeper insight into the ecological role of the extracellular levansucrase and the formed levan.

## Materials and methods

### Levansucrase recovery and levan production at different pH and sucrose concentrations

**Fig. 1:**
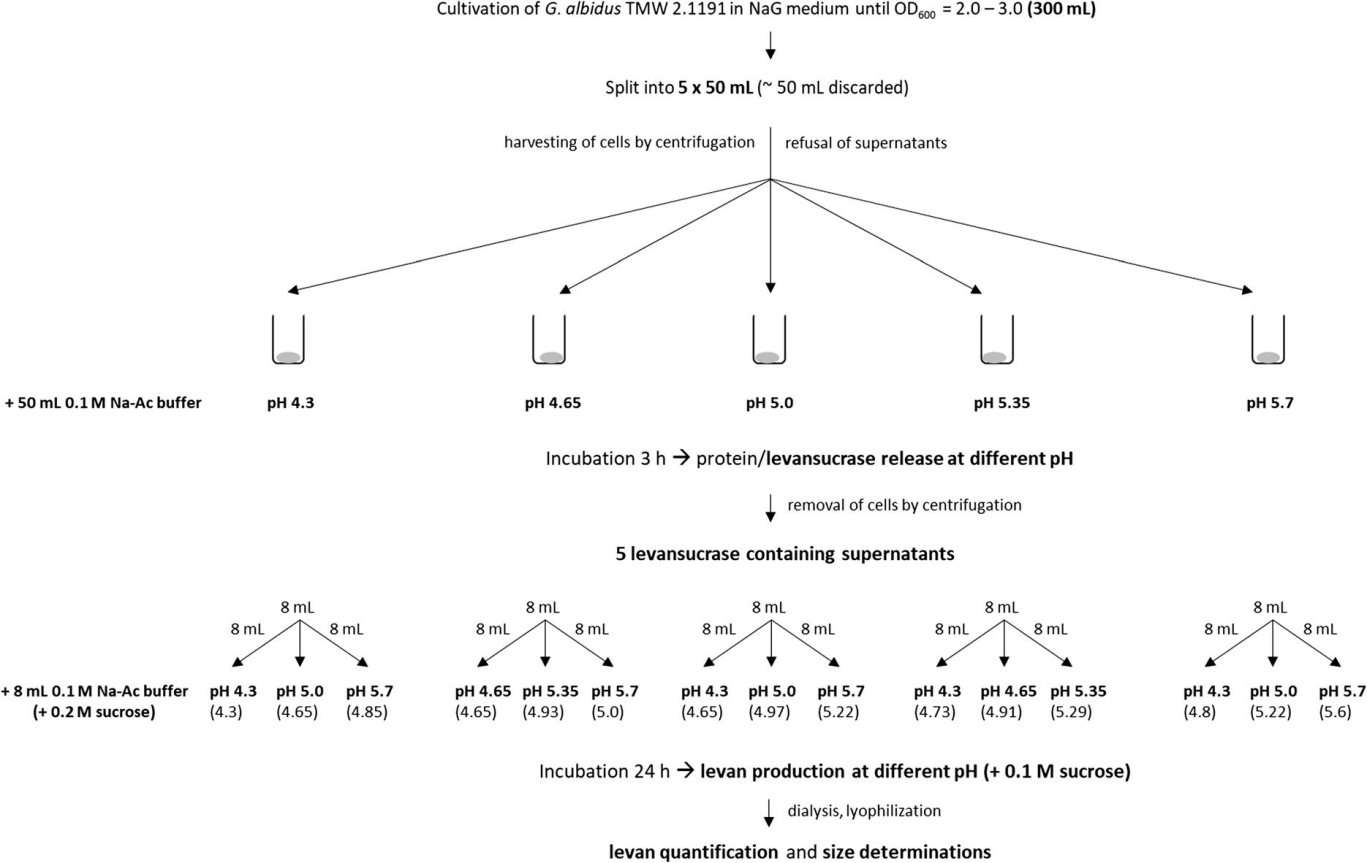
Workflow for the recovery of levansucrase containing supernatants and for the subsequent levan production at different pH (chapter 3.2). The pH values in brackets indicate the determined final production pH after mixing of the respectively used buffers. Three independently grown cell cultures (300 mL; OD 2.2, 2.58, 2.84) were prepared and handled as depicted. The obtained results about amounts and sizes of the recovered levans as well as contained volumetric levansucrase activities (in total 15 samples per cell culture: 5 release pH × 3 production pH) were finally compared among each other (Fig. 3 and Supplementary File 1)

*Gluconobacter* (*G.*) *albidus* TMW 2.1191 isolated from water-kefir (Jakob et al. 2012a; Ua-Arak et al. 2017a) was incubated in Erlenmeyer flasks, which were filled with 10% liquid medium relative to the total volume of the flask to facilitate aerobic growth on a rotary shaker (200 rpm). A general overview of the experimental steps for levansucrase recovery and levan production at different pH is depicted in Fig. 1. The NaG-medium used for precultivation of *G. albidus* contained 20 g/L sodium gluconate, 3 g/L yeast extract, 2 g/L peptone, 3 g/L glycerol, 10 g/L mannitol, 3 g/L glucose (initial pH adjusted to 6.0). The optical densities (ODs) of the fermented NaG media were determined at 600 nm in a Novaspec Plus spectrophotometer (Amersham Biosciences, Germany). The number of cells in liquid culture media was determined as colony forming units (CFU) on solid NaG agar (15 g/L) media in duplicates. Harvested cells from liquid cultivations in NaG medium were resuspended in 0.1 M sodium-acetate (Na–Ac) buffers, which had been adjusted to five different pH values (4.3–5.7). For investigation of the impact of the levansucrase-release pH on levan production at different pH (3.1), the levansucrase-containing supernatants were diluted 1:1 (8 mL + 8 mL) with three Na–Ac buffers of different pH, respectively, which had been supplemented with 0.2 M sucrose for subsequent levan production. The determined final production pH values per levansucrase release pH are shown in brackets in Fig. 1. This experiment was performed three times using three independently grown main cultures (300 mL, Fig. 1), which had been grown to an optical density OD (600 nm) of 2.2, 2.58 and 2.84, respectively. For investigation of the impact of the initial sucrose concentration on levan production and levan sizes (3.2), three independently grown cell cultures (OD 2.62; 2.77; 3.0) were used. In contrast to the experiment described above, the five levansucrase containing supernatants recovered at pH 4.3, 4.65, 5.0, 5.35 and 5.7 (Fig. 1) were diluted 1:1 with four Na–Ac buffers adjusted to the same pH used for levansucrase release (e.g. 4.3 + 4.3), but containing four different sucrose concentrations (0.1/0.2/0.4/0.8 M). For levan production, the solutions were statically incubated for 24 h at 30 °C in both experimental series. The levan samples were dialyzed (MWCO: 3.5 kDa) against ddH_2_O (4 °C; 48 h) for removal of sugars and fructooligosaccharides < 3.5 kDa, lyophilized and weighed.

### Separation and size determinations of levans by AF4-MALLS

The radii distributions of the produced high molecular weight levans were determined by asymmetric flow field-flow fractionation (AF4; Eclipse Dualtec, Wyatt Technology, USA) coupled to multi-angle laser light scattering (MALLS) (Dawn EOS: levans produced at different pH values; DAWN Heleos II: levans produced at different sucrose concentrations; Wyatt Technology, USA). The lyophilized levans were dissolved in ddH_2_O to a final concentration of 0.1 mg/mL. 100 µl of the respective sample (10 µg) were then injected into the separation channel, equipped with a 10 kDa cellulose membrane (Nadir regenerated cellulose). Levan separations were performed using a detector-flow rate of 1 mL/min and a cross-flow gradient of 3 to 0.1 mL/min over 15 min, followed by 15 min of a steady cross flow of 0.1 mL/min. The obtained chromatograms were analyzed with the software ASTRA 6 (Wyatt Technologies, Germany) using the integrated particle mode and the sphere model. The accuracy and reproducibility of levan separations on the used cellulose membranes was randomly checked by either remeasuring of levan samples or measuring of bovine serum albumin (in this case injection and separation of 50 µg using a constant crossflow of 5 mL/min for 25 min).

### Protein separation, visualisation and identification

For separation of proteins recovered in buffers, SDS gels (10%) were prepared using the materials of a Mini-PROTEAN set (BioRad, Germany). Silver and Coomassie stainings were performed for visualization of proteins. Protein concentrations were determined in triplicate in 96 well plates using the Bradford assay (Sigma Aldrich, Germany). The buffers used for levansucrase release (8 mL; Fig. 1) were concentrated 80x (resuspension of proteins in 100 µl ddH_2_O after lyophilization of buffers) before SDS-PAGE (and Bradford analysis), as no bands could be visualized without concentration of the samples. For verification of the levansucrase, the stained protein of the expected size (~ 48 kDa) was excised from the SDS gel and sent to the “Zentrallabor für Proteinanalytik” (ZfP, Ludwig-Maximilians-Universität München). Upon tryptic digestion and modification of the respective proteins for proper separation and mass spectra generation by LC–MS/MS, the obtained mass spectra were compared with in silico tryptically digested proteins of the domain *Eubacteria* (deposited at NCBI) using the Matrix Science Mascot software (Perkins et al. 1999). Additionally, the “Mascot generic format (.mgf) formatted” files derived from LC–MS/MS analysis, were processed to peptide sequences with PepNovo (Frank and Pevzner 2005; Frank et al. 2005, 2007) and “blasted” against available proteoms of AAB, as also described by Behr et al. (Behr et al. 2007), to confirm the obtained Mascot search results.

### Determination of levansucrase activities

The mean activities (24 h) of the native levansucrases used for production and determination of sizes of levans were calculated by determination of enzymatically released sugars via HPLC analysis using a Rezex RPM ion-exclusion column (Phenomenex, Germany) coupled to a refractive index (RI) detector (Gynkotek, Germany). The water flow (mobile phase) was kept constant at 0.6 mL/min during each run, separations were performed at 85 °C. Calibration curves were established using the standards glucose and fructose in different concentrations (1–100 mM). The released glucose was used for calculation of the overall activity, while the detected fructose was (additionally) used for calculation of the hydrolysis (concentration fructose) and transfructosylation activities (concentration glucose–concentration fructose) (Tieking et al. 2005). Volumetric activities of levansucrases are expressed in Units (U), which are defined as µmol/mL (protein sample) * min. In doing so it is assumed that no other sucrose, glucose and fructose converting activities affecting the activity calculations were present in the crude extracts containing the natively released or heterologously expressed (below) levansucrase.

For comparison of the kinetics of the *G. albidus* TMW 2.1191 levansucrase at different pH and sucrose concentrations (Supplementary File 2), the cloned levansucrase gene was heterologously expressed in *Escherichia* (*E.*) *coli* Top 10 (Jakob et al. 2012a) by induction with arabinose (1 mM, 25 °C, o/n, 200 rpm) according to the instructions of the pBAD cloning/expression manual (Invitrogen, Germany). After harvesting of the cells by centrifugation (30 mL culture; 10 min; 5000×*g*), the cells were resuspended in 1 mL Na–Ac buffer (pH 5), as preliminary experiments had revealed that the recombinant levansucrase drastically lost its activity at pH 7 (compare also supplementary File 2). The cells were subsequently lysed by sonication and the insoluble debris was removed by centrifugation (10,000×*g*). For activity assays, the obtained levansucrase-containing supernatants were again diluted 1:30 in 0.1 M sodium acetate buffer (pH 5.0) and directly applied, as the enzyme could not be purified at pH 5.0 by Ni–NTA affinity chromatography according to the manufacturers´ instructions (performed at alkaline pH (Spriestersbach et al. 2015)). Non-induced *E. coli* Top 10 cultures/lysates thus served as control to exclude any intrinsic sucrose related activities in the *E. coli* lysates. A reaction assay consisting of 100 µl sucrose (0.025 M–1.6 M), 50 µl 0.4 M citric acid/sodium citrate buffer (pH 3.0–4.0) or Na–Ac buffer (pH 4.3–5.7) or NaH_2_PO_4_/Na_2_HPO_4_ buffer (pH 6.0–7.0), 45 µl ddH_2_O and 5 µl of the enzyme dilution was incubated in a water bath at 30 °C for 1 h. The same volume (200 µl) of 0.25 M NaOH was then added and mixed thoroughly to stop the enzymatic reaction. 10 µl of each preassay sample was transferred in triplicates to a Microtest plate (96 wells, Sarstedt) and mixed with 200 µl ddH_2_O, 10 µl buffer 1 and 10 µl buffer 2 from the d-Fructose/d-Glucose Assay Kit (Megazyme, Ireland). After 3 min the initial absorbance was measured with a FLUOstar Omega microplate reader (BMG Labtech, Germany). 10 µl of solution 3 (included in kit) were then added, the microplate was shaken for further 20 s and the second absorbance was measured after 8 min. Afterwards, 10 µl of solution 4 were added and the microplate was again shaken for 20 s. The final absorbance was measured after 10 min. The glucose and fructose concentrations were calculated via the law of Lambert–Beer, respectively. For pH 3.0 and pH 3.5, sucrose hydrolysis was observed in the control samples (without enzyme solution), most likely due to spontaneous acidic hydrolysis at low pH. In these cases, additional controls were applied for all substrate concentrations and included in the levansucrase activity calculations.

### Statistical analysis

Data depicted in Figs. 3, 5 and Supplementary File 2 are expressed as mean values including standard deviations (SD) derived from three independent experiments, respectively. The origin of these data is further specified in the respective results sections and figure captions. The bilateral homoscedastic *t* test was used to describe significant differences at 5% significance level (*p* < 0.05), respectively.

## Results

### Analysis of buffer supernatants recovered at different pH

**Fig. 2:**
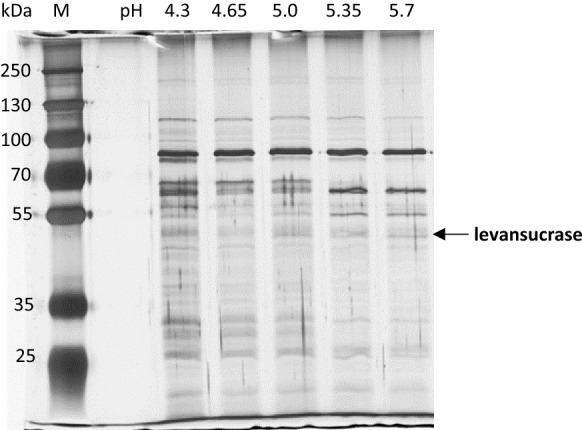
SDS-PAGE of levansucrase containing supernatants recovered at pH 4.3, 4.65, 5.0, 5.35 and 5.7. M: marker

The buffer supernatants obtained after 3 h of incubation at pH 4.3, 4.65, 5.0, 5.35 and 5.7 (Fig. 1) were analyzed regarding their respective protein contents and the presence of the levansucrase. The protein amount was always below the detection limit of the used assay (0.1 mg/mL), even if the buffer supernatants were concentrated 80x before analysis (8 mL → 0.1 mL). Therefore, a total protein amount < 0.01 mg/mL was present in all samples. However, after 80× concentration of the samples and subsequent SDS-PAGE, the extracellular proteins could be visualized by silver staining. On the contrary, no proteins could be visualized by Coomassie staining at any tested condition. Similar profiles were detected at the tested pH values 4.3/4.65/5.0. Few differences could be observed in regard to the presence of certain proteins at 5.35 and 5.7 (Fig. 2). The levansucrase monomer exhibiting a putative size of ~ 48 kDa (NCBI accession number: AQS91558) was cut out of the gel (pH 5.7) and verified by mass-based peptide sequencing as described previously by Jakob (2014).

### Amounts and radii distributions of levans produced at different pH initially used for levansucrase recovery

**Fig. 3:**
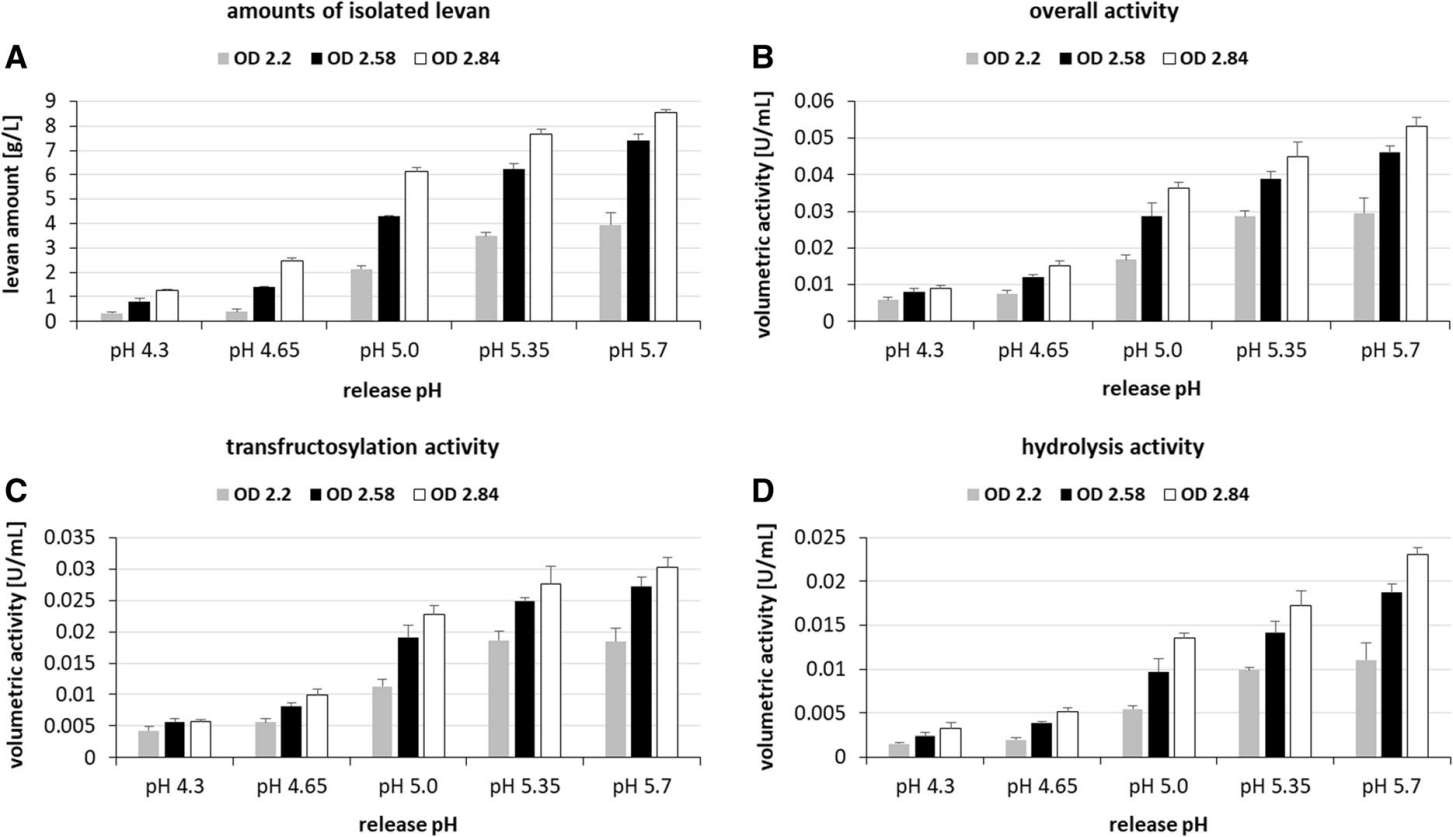
Produced levan amounts (**A**) and the corresponding volumetric overall (**B**), transfructosylation (**C**) and hydrolysis (**D**) activities determined after 24 h of levan production at different pH using three different main cultures (OD 2.2, OD 2.58, OD 2.84). Mean values (n = 3) including standard deviations (SD) were calculated from three different production pH per release pH/used cell culture (Supplementary File 1), respectively

The collected enzyme-containing supernatants were used to produce levan at 0.1 M sucrose and three different pH values per release pH (Fig. 1). This approach was repeated in triplicate using three main cultures (300 mL) of *G. albidus* TMW 2.1191 grown to OD_600_ = 2.2, 2.58, 2.84, respectively, which were split (5 × 50 mL) for enzyme release at different pH, respectively (Fig. 1). The CFU/mL increased with increasing OD (OD 2.2: 8.5 ± 0.25 * 10^8^; OD 2.58: 1.05 ± 0.24 * 10^9^; OD 2.84: 1.17 ± 0.18 * 10^9^). After levan quantification and determination of the respective overall, transfructosylation and hydrolysis activities it was observed, that the amounts of isolated levan and the corresponding activities were in a similar range per release pH (Supplementary File 1A–F). Therefore, it could be assumed that the final production pH was not decisive within the tested pH range, if the same enzyme solution recovered at a certain pH condition was used for levan production. The three values obtained per release pH were thus averaged to compare the impact of the initial cell density and the release pH on the levan formation (Fig. 3).

**Fig. 4:**
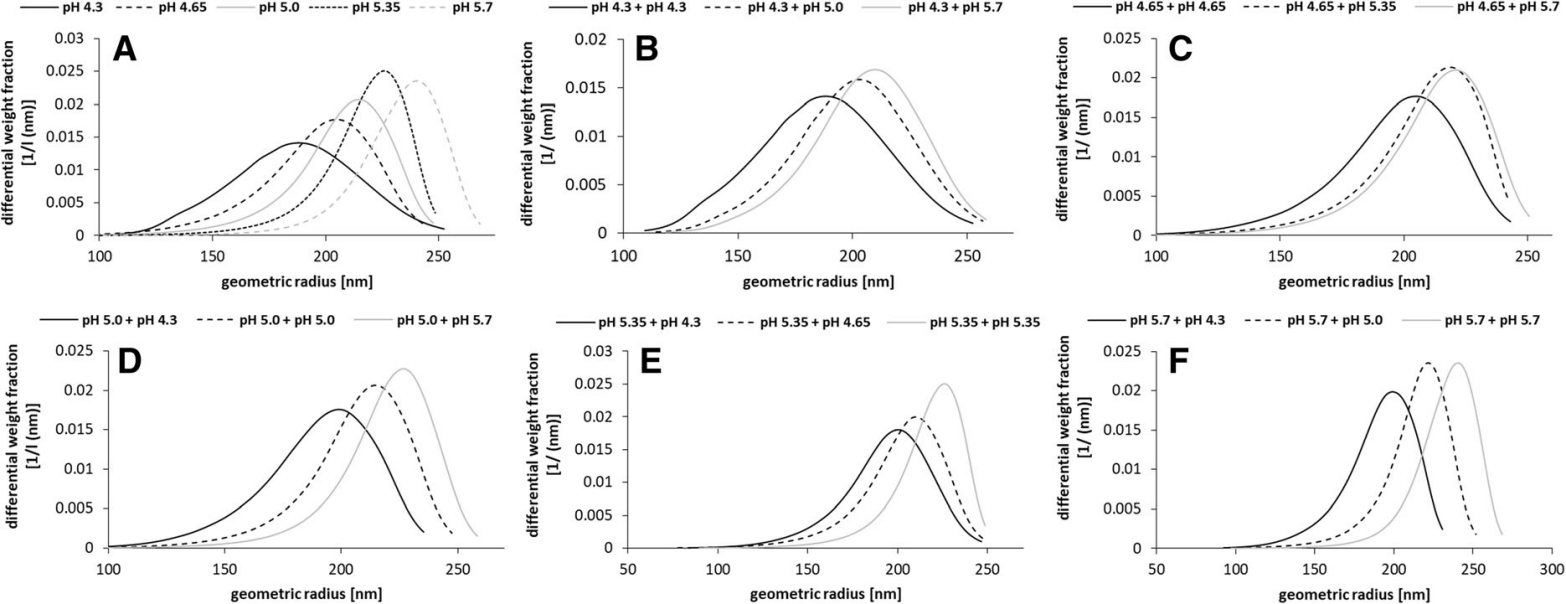
Differential weight distributions of geometric radii of levans produced at different pH. The respective radii distributions of levans produced at the pH of levansucrase release are depicted in (**A**) and in (**B**–**F**) of the three different production pH per release pH of the levansucrase: pH 4.3 (**B**), 4.65 (**C**), 5.0 (**D**), 5.35 (**E**) and 5.7 (**F**). The experimentally determined production pH values derived from the respective buffer mixtures are depicted in brackets in Fig. 1. Data are exemplarily shown for the cell culture OD (600 nm) = 2.58 of *G. albidus* TMW 2.1191 and were highly similar for OD 2.2 and 2.84 (data not shown), respectively

The isolated levan amounts were significantly higher (*p* < 0.05), if a higher initial cell density had been applied (Fig. 3A). Moreover, the levan amounts significantly increased with rising production pH, respectively, with two exceptions at OD 2.2 (pH 4.35 compared to pH 4.65 and pH 5.35 compared to pH 5.7 → *p* > 0.05). A similar trend was observed upon comparison of the calculated volumetric activities, which were accordingly higher, if a higher cell density and release pH had been initially applied (Fig. 3B–D). The size distributions of the levan molecules were shifted per release pH, even if they had been produced in comparable amounts at different pH values using constant protein amounts (Fig. 4). The respective peak maxima, which are representative for the majority of molecules exhibiting a certain size, were generally shifted to higher geometric radii, if the levan had been produced at higher pH (Fig. 4A–F).

### Amounts and radii distributions of levans produced at different pH and sucrose concentrations

**Fig. 5:**
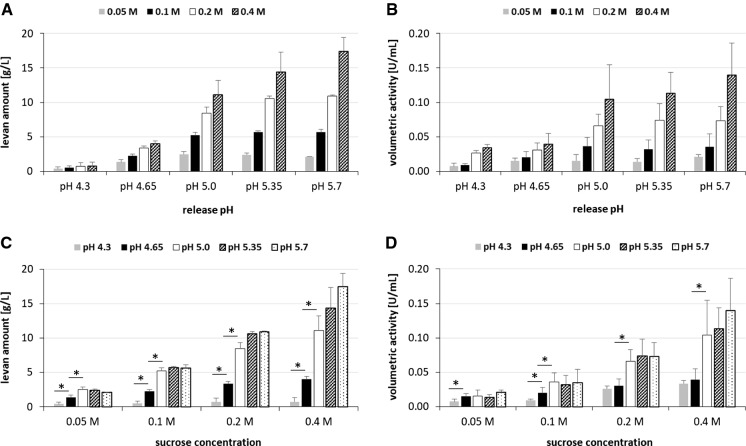
Produced levan amounts **A**, **C** at the pH of levansucrase release (pH 4.3, 4.65, 5.0, 5.35, 5.7) using four different sucrose concentrations (0.05, 0.1, 0.2, 0.4 M) and the corresponding volumetric activities **B**, **D** determined after 24 h of levan production using three different main cultures (OD 2.63, OD 2.77, OD 3.0). Mean values (n = 3) including standard deviations (SD) were calculated from singly detemined values per cell culture and specific condition (pH and sucrose concentration), respectively. Stars indicate significant differences (*p* < 0.05) between the compared conditions (marked by lines)

**Fig. 6:**
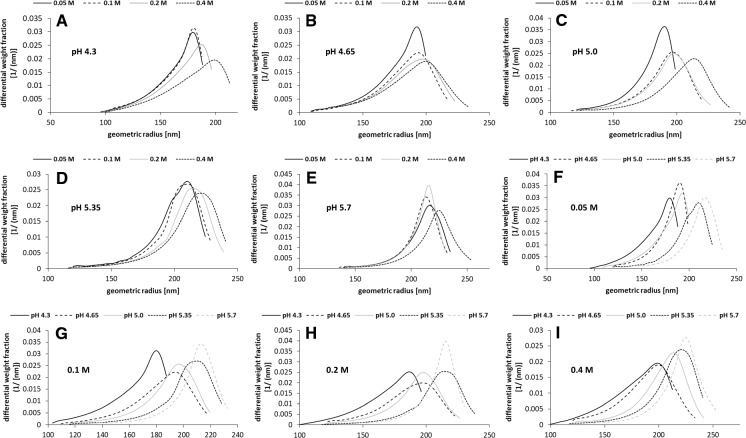
Differential weight distributions of geometric radii of levans produced at different pH (pH 4.3, 4.65, 5.0, 5.35, 5.7) and sucrose concentrations (0.05, 0.1, 0.2, 0.4 M). The experimentally determined production pH values derived from the respective buffer mixtures are depicted in brackets in Fig. 1. Data are exemplarily shown for the cell culture OD (600 nm) = 2.77 of *G. albidus* TMW 2.1191 and were highly similar for OD 2.63 and 3.0 (data not shown), respectively

Additional experiments using the heterologously expressed levansucrase of *G. albidus* TMW 2.1191 revealed that the enzyme exhibits comparable activities and Michaelis–Menten kinetics within a broad pH range (Supplementary File 2). Therefore, the influence of the sucrose concentration on the produced levan amounts and sizes was additionally investigated. For this purpose, three additional cell cultures (each 300 mL; OD 2.63: 1.33 ± 0.16 CFU/mL, OD 2.77: 1.42 ± 0.05 CFU/mL, OD 3.0: 1.575 ± 0.025 * 10^9^ CFU/mL) were used for recovery of levansucrase-containing supernatants at pH 4.3, 4.65, 5.0, 5.35 and 5.7 according to the workflow depicted in Fig. 1. After removal of cells, the supernatants were used to produce levan at four different sucrose concentrations (0.05, 0.1, 0.2, 0.4 M) and at the pH of levansucrase release, respectively (20 samples per cell culture). The produced levans were quantified once per cell culture and averaged among the three cell cultures (Fig. 5A). Moreover, the overall activities were determined in all samples (Fig. 5B). Except for pH 4.3, significant higher amounts of levan were isolated per release pH, if a higher initial sucrose concentration had been applied (Fig. 5A). This finding was confirmed via calculation of the respective overall activities, which increased per release pH using higher sucrose concentrations (Fig. 5B). The levan amounts significantly increased with rising release pH of the levansucrase using equal initial sucrose concentrations between pH 4.3/4.65 and pH 4.65/5.0 (Fig. 5C). Between pH 4.65/5.0 this was also confirmed by the calculated overall activities (Fig. 5D). The levan sizes slightly increased per release pH using higher initial sucrose concentrations (Fig. 6A–E). Moreover, the levan sizes increased with rising release pH using equal initial sucrose concentrations (Fig. 6F–I).

## Discussion

Previous studies revealed that the spherical high molecular weight levan molecules produced by *G. albidus* TMW 2.1191 are functionally diverse regarding their hydrocolloid and rheological properties depending on their molecular size (Hundschell et al. 2019, 2020; Jakob et al. 2012b, 2013; Ua-Arak et al. 2016, 2017b). Moreover, the production pH during batch fermentation is crucial for the size distributions of these levans (Hundschell et al. 2020; Ua-Arak et al. 2017a) as also shown in the present work (Figs. 4, 6). However, little is known about the influencing factors of levansucrase release as well as of levansucrase activity, both of which are crucial for the efficiency of the complex production process of polydisperse levan taking place under continuously changing conditions. By application of the developed buffer system it was confirmed that the levansucrase is constitutively expressed by *G. albidus* TMW 2.1191 (without induction by its substrate sucrose) as reported for some dextransucrases secreted by water kefir LAB (Bechtner et al. 2019; Schmid et al. 2019). Higher volumetric levansucrase activities were detected in buffer supernatants at higher release pH (Fig. 3). The higher productivity towards levan formation at higher release pH could be due to comparatively higher levansucrase amounts released at higher pH. This view is supported by the fact that the use of the same crude enzyme preparation for levan production yielded comparable levan amounts (Supplementary File S1). Moreover, higher volumetric activities were determined in buffers incubated with higher cell densities (Fig. 3) indicating that more levansucrase was released by a higher number of metabolic active cells. However, the continuous increase in productivity with rising release pH could not be verified in the second experimental series focusing on the impact of the sucrose concentration on levan formation (3.3; Fig. 5), in which significant increases in levan amounts and volumetric overall activities could solely be observed between pH 4.3/4.65 or rather pH 4.65/5.0 (Fig. 5). Hence, in addition to the environmental pH, the cell densities and/or growth phase of the levan producing cultures influenced the levan formation, as higher cell densities were applied in the second experimental series (3.3). A growth phase-dependent expression of levansucrases was also reported for the enteric bacterium *Rahnella aquatilis* (Seo et al. 2002), at which it has to be considered that expression and secretion/release of sucrases are in fact independent processes. For instance, the water kefir isolate *Lactobacillus hordei* TMW 1.1822 releases its sucrose-converting dextransucrases in similar amounts into the environment in dependence of sucrose, but independently of the applied environmental release pH while accumulating the dextransucrases within the cell independent of sucrose (Bechtner et al. 2019; Schmid et al. 2019). In case of *Lactobacillus hordei* TMW 1.1822 it was further observed that the release pH affects the mean activity/productivity of the dextransucrase at different pH. This probably resulted from the concomitantly increased stability of the dextransucrase towards its denaturation at non-optimum pH, if it had been initially recovered actively at its approximate optimum pH. A similar feature is unlikely for the levansucrase released by *G. albidus* TMW 2.1191, as it appears to be comparably active and productive towards high molecular weight levan production over a broad pH range (Supplementary Files 1 + 2). This suggests that this type of levansucrase is structurally adapted to changes in the extracellular pH, which naturally result from gluconic (and acetic) acid production by *G. albidus* TMW 2.1191. Noticeably, *Gluconobacter* levansucrases are next related to those of *Zymomonas mobilis* (Jakob et al. 2019), whose expressed levansucrase monomers self-assemble to ordered oligomers/microfibrils at low pH and high ionic strength (Goldman et al. 2008). A similar structural adaptation towards a stable activity in the acidic environment could thus be assumed for the levansucrase of *G. albidus* TMW 2.1191.

In contrast to the overall glucose release and produced levan amounts, the levan sizes, which are decisive for the macromolecular properties of levan (Hundschell et al. 2019; Jakob et al. 2013), were influenced by the production pH (Figs. 4 + 6). This could be due to different substrate saturations of the levansucrases (Fig. 6 + Supplementary File 2), an additional expressed β-fructosidase (WP077802344) (Brandt et al. 2017; Jakob et al. 2019) and/or the possible intrinsic levanase activity (Méndez-Lorenzo et al. 2015) of the levansucrase at certain conditions. The presented experimental approach could hence be used to control and trigger the sizes of globular levan molecules. On the other hand it remains unclear if the variably influenced processes of levan biosynthesis (e.g. by a continuously changing pH) can be naturally controlled by microbes for production of levan fractions, which exhibit specific properties upon occupation of sucrose-rich niches and biofilm formation. The comparable total activity of this levansucrase over a broad acidic pH range and release of presumably more levansucrase at higher pH may, however, help *G. albidus* TMW 2.1191 to efficiently release glucose (and fructose by hydrolysis) from sucrose. Glucose can be either directly incompletely oxidized to gluconic acid or intracellularly metabolized to pyruvate usually leading to the additional extracellular accumulation of acetic acid (Peters et al. 2013). Consequently, a stronger accumulation of acids via extracellular sucrose utilization at comparatively higher pH would allow quick energy generation and efficiently prevent the growth of competing, non-acid tolerant microbes. The more efficient release of constitutively expressed levansucrases by *G. albidus* TMW 2.1191 at higher pH could hence be considered as an adapted physiological feature for targeted colonization of sucrose-containing habitats.

## Electronic supplementary material

Below is the link to the electronic supplementary material.

Supplementary File 1: Produced levan amounts at different pH and initial OD (600 nm) of the cell culture (OD 2.2, 2.58, 2.84) of *G. albidus* TMW 2.1191 (A–C) and the corresponding volumetric activities (D–F) determined after levan production. The experimentally determined production pH values derived from the respective buffer mixtures are depicted in brackets in Fig. 1. (PPTX 159 kb)

Supplementary File 2: Volumetric activities of the heterologously expressed levansucrase of *G. albidus* TMW 2.1191 at different pH values and sucrose concentrations. Black dot: overall activity; white square: hydrolysis activity; white rhomb: transfructosylation activity. Each assay (pH 3-0–pH 7.0; 0.05–0.8 M sucrose) was performed thrice using three different protein stocks obtained from three independently grown *E. coli* Top 10 cell cultures, which had been induced by addition of 1 mM arabinose, respectively. The depicted data are mean values (n = 3) including standard deviations (SD) derived from the three independently performed assays. (PPTX 7731 kb)

## References

[CR1] Arrieta J (1996). Molecular characterization of the levansucrase gene from the endophytic sugarcane bacterium Acetobacter diazotrophicus SRT4. Microbiology.

[CR2] Bechtner J, Wefers D, Schmid J, Vogel RF, Jakob F (2019). Identification and comparison of two closely related dextransucrases released by water kefir borne *Lactobacillus hordei* TMW 1.1822 and Lactobacillus nagelii TMW 1.1827. Microbiology.

[CR3] Behr J, Israel L, Gänzle MG, Vogel RF (2007). Proteomic approach for characterization of hop-inducible proteins in Lactobacillus brevis. Appl Environ Microbiol.

[CR4] Brandt JU, Jakob F, Geissler AJ, Behr J, Vogel RF (2017). Multiple genome sequences of heteropolysaccharide-forming acetic acid bacteria. Genome Announc.

[CR5] Deppenmeier U, Ehrenreich A (2009). Physiology of acetic acid bacteria in light of the genome sequence of Gluconobacter oxydans. J Mol Microbiol Biotechnol.

[CR6] Dogsa I, Brloznik M, Stopar D, Mandic-Mulec I (2013). Exopolymer diversity and the role of levan in *Bacillus subtilis* biofilms. PLoS ONE.

[CR7] Fels L, Jakob F, Vogel RF, Wefers D (2018). Structural characterization of the exopolysaccharides from water kefir. Carbohydr Polym.

[CR8] Frank A, Pevzner P (2005). PepNovo: de novo peptide sequencing via probabilistic network modeling. Anal Chem.

[CR9] Frank A, Tanner S, Bafna V, Pevzner P (2005). Peptide sequence tags for fast database search in mass-spectrometry. J Proteome Res.

[CR10] Frank AM, Savitski MM, Nielsen ML, Zubarev RA, Pevzner PA (2007). De novo peptide sequencing and identification with precision mass spectrometry. J Proteome Res.

[CR11] Goldman D, Lavid N, Schwartz A, Shoham G, Danino D, Shoham Y (2008). Two active forms of Zymomonas mobilis levansucrase an ordered microfibril structure of the enzyme promotes levan polymerization. J Biol Chem.

[CR12] Hundschell CS, Jakob F, Wagemans AM (2019) Molecular weight dependent structure and polymer density of the Exopolysaccharide Levan. arXiv preprint arXiv:190907737 10.1016/j.ijbiomac.2020.06.019 32512087

[CR13] Hundschell CS, Braun A, Wefers D, Vogel RF, Jakob F (2020). Size-dependent variability in flow and viscoelastic behavior of levan produced by Gluconobacter albidus TMW 2.1191. Foods.

[CR14] Jakob F (2014) Novel fructans from acetic acid bacteria. Technische Universität München

[CR15] Jakob F, Meißner D, Vogel RF (2012). Comparison of novel GH 68 levansucrases of levan-overproducing Gluconobacter species. Acetic Acid Bacteria.

[CR16] Jakob F, Steger S, Vogel RF (2012). Influence of novel fructans produced by selected acetic acid bacteria on the volume and texture of wheat breads. Eur Food Res Technol.

[CR17] Jakob F, Pfaff A, Novoa-Carballal R, Rübsam H, Becker T, Vogel RF (2013). Structural analysis of fructans produced by acetic acid bacteria reveals a relation to hydrocolloid function. Carbohydr Polym.

[CR18] Jakob F, Quintero Y, Musacchio A, Estrada-de los Santos P, Hernández L, Vogel RF (2019). Acetic acid bacteria encode two levansucrase types of different ecological relationship. Environ Microbiol.

[CR19] Koczan JM, McGrath MJ, Zhao Y, Sundin GW (2009). Contribution of Erwinia amylovora exopolysaccharides amylovoran and levan to biofilm formation: implications in pathogenicity. Phytopathology.

[CR20] Korakli M, Pavlovic M, Gänzle MG, Vogel RF (2003). Exopolysaccharide and kestose production by Lactobacillus sanfranciscensis LTH2590. Appl Environ Microbiol.

[CR21] Laue H, Schenk A, Li H, Lambertsen L, Neu TR, Molin S, Ullrich MS (2006). Contribution of alginate and levan production to biofilm formation by Pseudomonas syringae. Microbiology.

[CR22] Méndez-Lorenzo L (2015). Intrinsic levanase activity of Bacillus subtilis 168 levansucrase (SacB). PLoS ONE.

[CR23] Öner ET, Hernández L, Combie J (2016). Review of levan polysaccharide: from a century of past experiences to future prospects. Biotechnol Adv.

[CR24] Perkins DN, Pappin DJ, Creasy DM, Cottrell JS (1999). Probability-based protein identification by searching sequence databases using mass spectrometry data. ELECTROPHORESIS: Int J.

[CR25] Peters B (2013). Deletion of pyruvate decarboxylase by a new method for efficient markerless gene deletions in Gluconobacter oxydans. Appl Microbiol Biotechnol.

[CR26] Schmid J, Bechtner J, Vogel RF, Jakob F (2019). A systematic approach to study the pH-dependent release, productivity and product specificity of dextransucrases. Microb Cell Fact.

[CR27] Semjonovs P, Shakirova L, Treimane R, Shvirksts K, Auzina L, Cleenwerck I, Zikmanis P (2016). Production of extracellular fructans by Gluconobacter nephelii P1464. Lett Appl Microbiol.

[CR28] Seo J-W (2002). Molecular characterization of the growth phase-dependent expression of the lsrA gene, encoding levansucrase of *Rahnella aquatilis*. J Bacteriol.

[CR29] Shih I-L, Yu Y-T, Shieh C-J, Hsieh C-Y (2005). Selective production and characterization of levan by *Bacillus subtilis* (Natto) Takahashi. J Agric Food Chem.

[CR30] Spriestersbach A, Kubicek J, Schaefer F, Block H, Maertens B (2015) Purification of His-tagged proteins. In: Methods in enzymology, vol 559. Elsevier, pp 1–15 10.1016/bs.mie.2014.11.003 26096499

[CR31] Támbara Y, Hormaza JV, Pérez C, León A, Arrieta J, Hernández L (1999). Structural analysis and optimised production of fructo-oligosaccharides by levansucrase from *Acetobacter diazotrophicus* SRT4. Biotech Lett.

[CR32] Tieking M, Gänzle MG (2005). Exopolysaccharides from cereal-associated lactobacilli. Trends Food Sci Technol.

[CR33] Tieking M, Ehrmann MA, Vogel RF, Gänzle MG (2005). Molecular and functional characterization of a levansucrase from the sourdough isolate *Lactobacillus sanfranciscensis* TMW 1.392. Appl Microbiol Biotechnol.

[CR34] Ua-Arak T, Jakob F, Vogel RF (2016). Characterization of growth and exopolysaccharide production of selected acetic acid bacteria in buckwheat sourdoughs. Int J Food Microbiol.

[CR35] Ua-Arak T, Jakob F, Vogel RF (2017). Fermentation pH modulates the size distributions and functional properties of Gluconobacter albidus TMW 2.1191 levan. Frontiers Microbiol.

[CR36] Ua-Arak T, Jakob F, Vogel RF (2017). Influence of levan-producing acetic acid bacteria on buckwheat-sourdough breads. Food Microbiol.

[CR37] Velázquez-Hernández M, Baizabal-Aguirre V, Bravo-Patiño A, Cajero-Juárez M, Chávez-Moctezuma M, Valdez-Alarcón J (2009). Microbial fructosyltransferases and the role of fructans. J Appl Microbiol.

[CR38] Xu Q, Yajima T, Li W, Saito K, Ohshima Y, Yoshikai Y (2006). Levan (β-2, 6-fructan), a major fraction of fermented soybean mucilage, displays immunostimulating properties via Toll-like receptor 4 signalling: induction of interleukin-12 production and suppression of T-helper type 2 response and immunoglobulin E production. Clin Exp Allergy.

