# Supplementary figures and images for: Insights into the pH-dependent, extracellular sucrose utilization and concomitant levan formation by Gluconobacter albidus TMW 2.1191
Source: Antonie Van Leeuwenhoek. 2020 Mar 4;113(7):863–73. doi: 10.1007/s10482-020-01397-3 (PMC7272483; doi:10.1007/s10482-020-01397-3)

## Slide 1
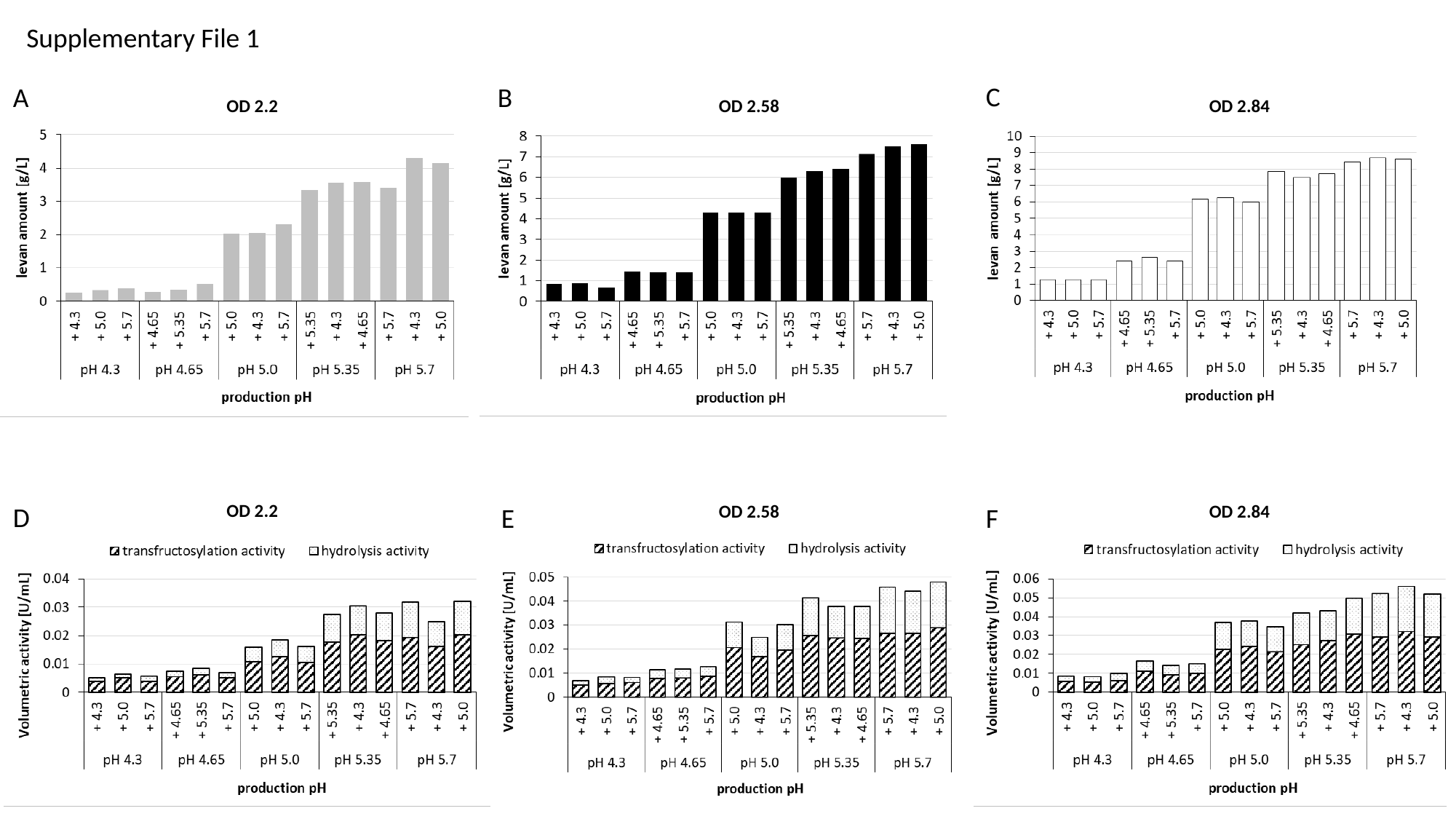

Supplementary File 1
C
A
B
OD 2.58
OD 2.84
OD 2.2
OD 2.2
OD 2.58
OD 2.84
D
E
F

Supplement: Supplementary file 1 — Produced levan amounts at different pH and initial OD (600 nm) of the cell culture (OD 2.2, 2.58, 2.84) of G. albidus TMW 2.1191 (A–C) and the corresponding volumetric activities (D–F) determined after levan production. The experimentally determined production pH values derived from the respective buffer mixtures are depicted in brackets in Fig. 1. (PPTX 159 kb) [file 10482_2020_1397_MOESM1_ESM.pptx]
