# Supplementary material for: Insights into the pH-dependent, extracellular sucrose utilization and concomitant levan formation by Gluconobacter albidus TMW 2.1191
Source: Antonie Van Leeuwenhoek. 2020 Mar 4;113(7):863–73. doi: 10.1007/s10482-020-01397-3 (PMC7272483; doi:10.1007/s10482-020-01397-3)
Supplement: Supplementary file 2 — Volumetric activities of the heterologously expressed levansucrase of G. albidus TMW 2.1191 at different pH values and sucrose concentrations. Black dot: overall activity; white square: hydrolysis activity; white rhomb: transfructosylation activity. Each assay (pH 3-0–pH 7.0; 0.05–0.8 M sucrose) was performed thrice using three different protein stocks obtained from three independently grown E. coli Top 10 cell cultures, which had been induced by addition of 1 mM arabinose, respectively. The depicted data are mean values (n = 3) including standard deviations (SD) derived from the three independently performed assays. (PPTX 7731 kb) [file 10482_2020_1397_MOESM2_ESM.pptx]

## Slide 1
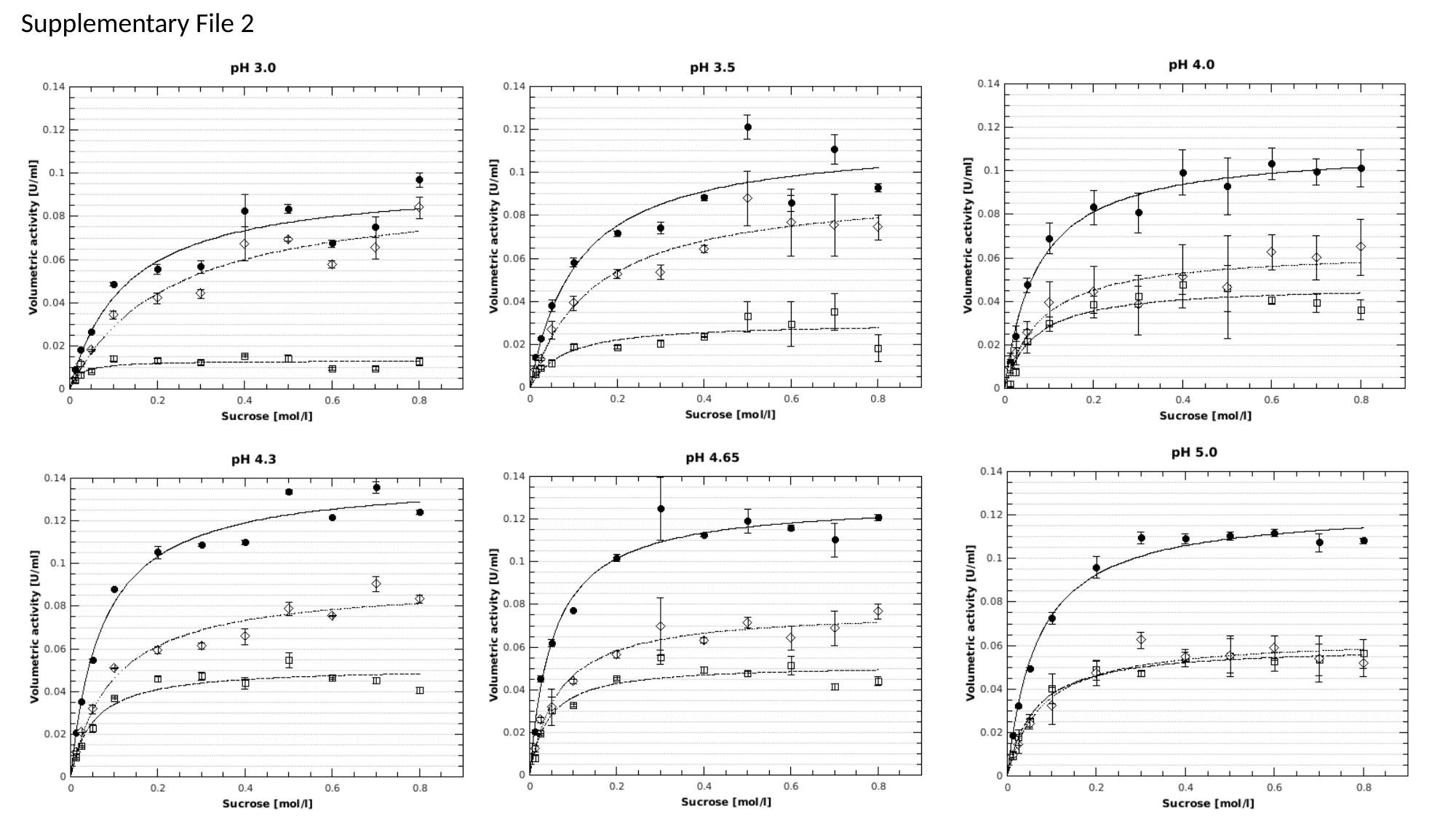

Supplementary File 2

## Slide 2
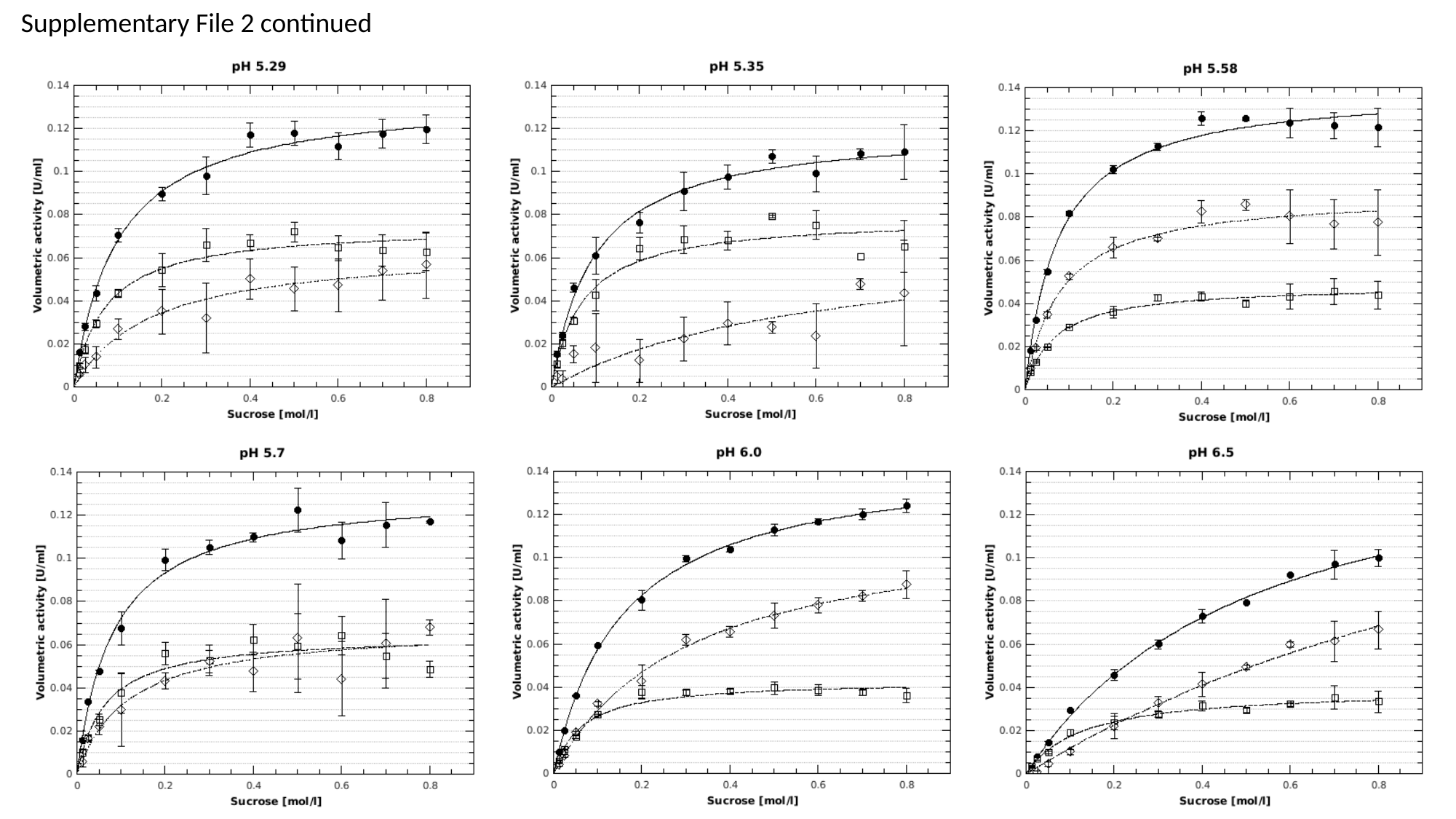

Supplementary File 2 continued

## Slide 3
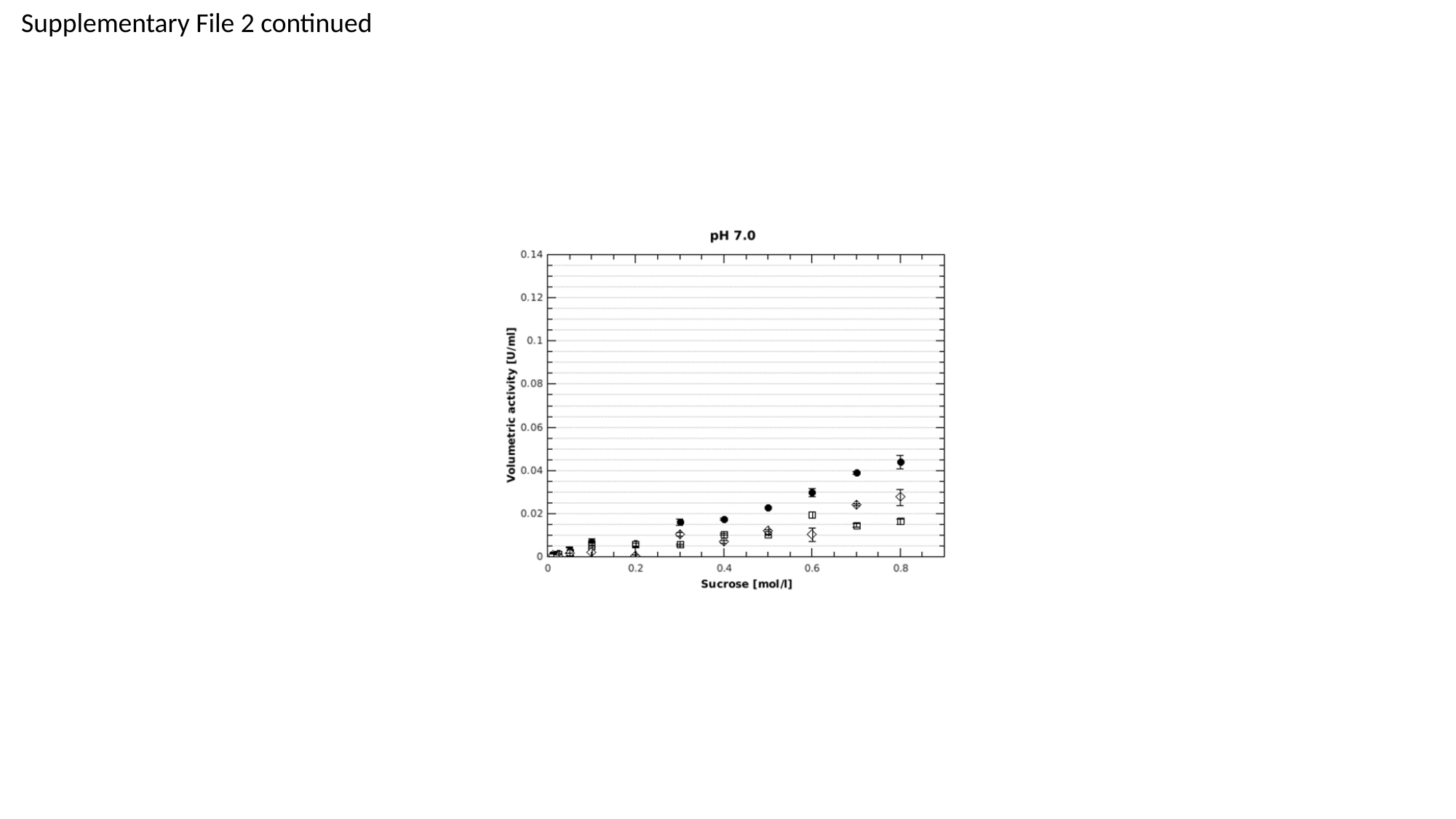

Supplementary File 2 continued
